# Barriers to Care Among LGBT Cancer Survivors: An Analysis of the All of Us Research Program

**DOI:** 10.3390/cancers18030398

**Published:** 2026-01-27

**Authors:** Madeline Brown-Savita, Jennifer M. Jabson Tree

**Affiliations:** Department of Public Health, Purdue University, West Lafayette, IN 47906, USA; brow1473@purdue.edu

**Keywords:** LGBT cancer survivors, healthcare utilization barriers, discrimination, minority stress, survivorship disparities, cluster analysis

## Abstract

**Background/Objectives**: Lesbian, gay, bisexual, and transgender (LGBT) cancer survivors face disproportionately high structural and psychosocial barriers to post-diagnosis care. However, heterogeneity within this population remains understudied. This study aimed to characterize healthcare utilization (HCU) barriers among LGBT cancer survivors, assess psychosocial vulnerabilities (discrimination, stress, and social support), and identify survivor subgroups at greatest risk for care disengagement. **Methods**: Data were drawn from the All of Us Research Program. A sample of 3502 LGBT cancer survivors was analyzed, including lesbian (*n* = 730), gay (*n* = 1285), bisexual (*n* = 1296), and transgender/gender expansive (TGE) (*n* = 209) individuals. HCU barriers were assessed using 21 binary indicators. Psychosocial measures included the Everyday Discrimination Scale, Perceived Stress Scale, and MOS Social Support Survey. Agglomerative hierarchical cluster analysis identified latent HCU barrier profiles. Differences across clusters and identity groups were assessed using ANOVA and chi-square tests, and multinomial logistic regression examined demographics, socioeconomic, and psychosocial predictors of cluster membership. **Results**: Three distinct HCU barrier clusters were identified: low (59.7%), moderate (27.8%), and high (12.5%). Bisexual and TGE survivors were disproportionately represented in the high-barrier cluster, which was characterized by widespread cost-related nonadherence, structural delays in care, and higher levels of perceived discrimination and stress. In adjusted models, bisexual identity, lower income, female sex assigned at birth, and higher discrimination and perceived stress were independently associated with increased odds of high-barrier cluster membership. **Conclusions**: Substantial heterogeneity exists in HCU barriers among LGBT cancer survivors. Bisexual and TGE survivors experience a concentrated burden of structural and psychosocial barriers to survivorship care, highlighting the relevance of targeted, data-driven approaches to reduce access inequities within this population.

## Simple Summary

Cancer survivorship does not look the same for all lesbian, gay, bisexual or transgender/gender expansive (LGBT) individuals, yet differences within the population are often overlooked. There is currently no clear way to describe how barriers to using healthcare cluster together among these survivors or how those barriers relate to stress, discrimination, and social support. This study used a data-driven approach and national data from the All of Us Research Program to address this gap. Cancer survivors were grouped based on shared patterns of healthcare access problems, and these groups were examined to understand differences in psychosocial experiences and LGBT identities. The goals were to identify common healthcare barriers, describe how stress, discrimination, and social support vary across survivor groups, and determine which factors are most strongly associated with greater barriers to care. This work helps clarify survivorship inequities and supports more targeted and equitable cancer care.

## 1. Introduction

Cancer survivorship presents unique and often overlooked challenges for individuals who identify as lesbian, gay, bisexual, transgender, or gender expansive (LGBT) [1,2]. Although estimates suggest that over one million LGBT individuals are living with a cancer diagnosis in the United States [3], this population remains underrepresented in survivorship research and underserved in clinical care. A growing body of evidence documents persistent disparities in healthcare access, including delays in diagnosis, under-treatment, and limited access to affirming care providers [4]. Structural barriers, such as affordability, transportation, and insurance coverage, intersect with experiences of marginalization to shape the post-treatment healthcare experiences of LGBT cancer survivors [5,6,7,8]. Despite increased recognition of these issues, the precise contours of survivorship inequities remain poorly understood, particularly for subgroups such as bisexual and transgender survivors who may experience distinct forms of stigma [9].

One key limitation of existing research is the tendency to treat LGBT populations as a monolithic group. This approach masks significant variation in experiences across sexual orientation and gender identity lines. For example, bisexual individuals often report invisibility and erasure both within LGBT spaces and in healthcare settings, particularly being overlooked or mischaracterized by providers and health systems [10,11]. Transgender and gender expansive (TGE) individuals frequently encounter provider discrimination, misgendering, disrespect, and outright refusal of care [1,12,13,14]. Aggregating data under a single LGBT umbrella limits our ability to identify subgroup-specific barriers and develop targeted interventions. Disaggregated analyses are essential to uncovering intra-group differences and addressing the nature of survivorship disparities. Understanding the distinct needs of bisexual and TGE cancer survivors, who face marginalization within both the broader society and the LGBT community, is critical for achieving equity in survivorship care.

Psychosocial factors play a central role in mediating access to care [4] and long-term outcomes for marginalized cancer survivors. Discrimination [15], chronic stress, and social isolation have been linked to worse physical and mental health among LGBT populations, in line with minority stress theory [16,17,18,19]. These experiences contribute not only to psychological distress but also to tangible barriers to care, such as avoiding healthcare settings, delaying treatment, or skipping medications [4]. While studies have begun to document these associations in general LGBT populations, there is a lack of research explicitly examining how psychosocial stressors shape healthcare utilization (HCU) barriers among LGBT cancer survivors. Recent population-based research has explicitly highlighted the lack of survivorship data for sexual and gender minority populations and identified a critical gap in understanding heterogeneity across subgroups defined by sexual orientation and gender identity [20]. Few studies use large national datasets to examine how psychosocial stressors and HCU barriers co-occur or cluster among LGBT cancer survivors.

Additionally, there is growing evidence that LGBT people’s experiences with psychosocial factors such as discrimination, social support, and stress relate differently to health outcomes than those of heterosexual and cisgender people [21,22,23]. Much of the published empirical literature concerning cancer survivorship among LGBT+ groups includes limited comparisons between sexual orientations and gender identities, and most often compares their health to heterosexual and cisgender samples. There are times when such comparisons are useful, such as when documenting disparities between minoritized groups and dominant groups. However, it is not necessary to conduct group comparisons in barriers to healthcare utilization among cancer survivors when trying to document and understand the associations between psychosocial factors and healthcare utilization barriers differently among LGBT cancer survivors specifically [24]. In fact, in these situations, comparing LGBT+ cancer survivors to heterosexual and cisgender cancer survivors can be problematic because it reinforces and perpetuates socially constructed cisgender and heteronormativity; the idea that heterosexuality is “the norm to which others are be compared” [25,26]. This methodological approach ignores the role of interpersonal, institutional, and internalized heterosexism that are different for LGBT people as they are for heterosexual and cisgender people. One possible solution is to compare LGBT+ cancer survivors to one another, rather than to heterosexual and cisgender cancer survivors, an approach which centers the experiences of LGBT+ cancer survivors. For example, much can be learned about associations between psychosocial factors, including social support and discrimination, and healthcare utilization barriers among and between LGBT+ cancer survivors without comparisons to heterosexual and cisgender survivors. This approach is aligned with calls to action to apply a critical lens to the consequence, purpose, and value of methodological choices, including the reference groups [26]. It also may assist in identifying specific psychosocial factors that could be used to improve care, policy and practice changes, and behavioral interventions for LGBT+ cancer survivors specifically.

To date, no typology exists to characterize how HCU barriers manifest across subgroups of LGBT cancer survivors. Cluster analysis offers a novel, data-driven method to identify meaningful subgroups based on shared healthcare access challenges [27,28]. Capturing heterogeneity in HCU burden can help clinicians, researchers, and policymakers better understand which survivors are most at risk of care disengagement and why. Such insight is essential for designing tailored interventions that address both structural and psychosocial drivers of inequity. This systemic classification can also shift the discourse from individual-level deficits to systemic patterns of marginalization and resilience across diverse identities.

To address these critical gaps, we analyzed data from the All of Us Research Program, a large and diverse national cohort, to characterize healthcare utilization barriers and psychosocial risk factors among LGBT cancer survivors. Using agglomerative hierarchical cluster analysis, we identified latent survivor subgroups based on patterns of HCU barriers and examined their associations with perceived discrimination, stress, and social support. Our objectives were to: (1) document the prevalence and distribution of cost-related and structural barriers to care across LGBT subgroups, (2) identify psychosocial disparities by sexual and gender identity, and (3) develop a typology of survivor profiles based on HCU burden. By applying a data-driven framework, this study advances the field’s understanding of survivorship disparities and provides an empirical foundation for more equitable care models.

## 2. Methods

### Data Source and Sample

Data was from the *All of Us* (AoU) Research Program’s Controlled Tier Dataset v8 (National Institutes of Health, Bethesda, MD, USA), available to authorized users on the Researcher Workbench [29]. The AoU Research Program is a diverse, longitudinal cohort study that collects survey, EHR, and biospecimen data from individuals across the United States [30]. For this secondary analysis, only data provided by LGBT cancer survivors were included. The sample included 3502 cancer survivors who identified as lesbian (*n* = 730), gay (*n* = 1285), bisexual (*n* = 1296), and transgender/gender expansive (*n* = 209).

## 3. Measures

### 3.1. Sexual Orientation and Gender Identity

Respondents self-reported their sexual orientation by answering a single question: “Which of the following best represents how you think of yourself?”. Included responses were Lesbian, Gay, or Bisexual. Respondents also self-reported their gender identity. To be included as a transgender/gender expansive identifying person, respondents had to answer transgender, non-binary, transgender man, transgender woman, genderqueer, genderfluid, gender variant, or two-spirit to either of the following questions: (1) “What terms best express how you describe your gender identity?” or (2) “Are any of these a closer description to your gender identity?” If sexual orientation and gender identity questions were skipped by respondents, they were not included in the current sample.

Within our analytic sample, 955 respondents did not respond to the gender identity question, 15 responded “unknown,” and 4559 skipped the item. For sexual orientation, 14,850 selected “none,” 8420 preferred not to answer, and 7021 skipped the item. Together, 33,761 individuals were not included. These individuals may have been cisgender, heterosexual, or LGBT, but because they did not provide explicit information, we cannot determine their identities.

### 3.2. Cancer Survivors

To be included in this sample, respondents had to have self-reported a personal history of any form of cancer. If respondents did not answer the cancer history question, they were not included in the sample. A total of 44,879 respondents did not report a history of cancer and were therefore excluded from the analytic sample. Among respondents who indicated a cancer history, 355 individuals were excluded because they did not provide cancer type.

Direct measures of time since cancer diagnosis were not available in the All of Us survey data. To partially account for timing-related differences in survivorship experiences, we incorporated age at cancer diagnosis as a proxy measure. Age group at diagnosis was included in descriptive analyses, bivariate comparisons, and multinomial logistic regression models to account for variation related to life stage at diagnosis, which may influence insurance coverage, healthcare access, and survivorship needs.

### 3.3. Healthcare Utilization Barriers

Healthcare Utilization Survey includes 21 binary variables to assess healthcare utilization barriers, affordability of care (e.g., “Couldn’t afford emergency care”), prescription access (e.g., “Took less medicine to save money”), and delays in care due to work, transportation, or caregiving responsibilities. All variables were recoded such that responses indicating a barrier were coded as 1 and all others as 0.

### 3.4. Psychosocial Factors

#### 3.4.1. Everyday Discrimination Scale

Perceived discrimination was measured using items from the Everyday Discrimination Scale (EDS), a widely used and extensively validated instrument of routine unfair treatment in daily life [31]. The EDS has demonstrated strong construct, convergent, and predictive validity across diverse populations, with higher scores consistently associated with psychological distress and adverse mental health outcomes [32,33]. Responses were coded on a 0 (“never”) to 5 (“almost every day”) scale and averaged to create a composite score, with higher values indicating greater perceived daily discrimination.

#### 3.4.2. Perceived Stress

Perceived stress was measured using the 4-item Perceived Stress Scale [34], a brief instrument designed to capture the extent to which individuals perceive their lives as unpredictable, uncontrollable, and overwhelming. Items were scored on a 0–4 Likert scale (“never” to “very often”) and averaged to produce a composite score, with higher values indicating greater perceived stress. The PSS-4 has demonstrated acceptable reliability and construct validity in large population-based studies and across diverse sociodemographic groups [35,36], and higher scores have been linked to adverse health outcomes [37].

#### 3.4.3. Social Support

Social support was assessed using the Medical Outcomes Study (MOS) Social Support Survey, a validated instrument measuring perceived availability of emotional/informational, tangible, affectionate, and positive social interaction support [38]. Items were scored on a 5-point scale and transformed to a standardized 0–100 score, with higher values indicating greater perceived support. The MOS Social Support Survey has demonstrated excellent reliability and validity across multiple populations, including studies involving LGBT and aging cohorts [39,40,41].

### 3.5. Statistical Analyses

Descriptive and summary statistics were calculated to describe the sample of LGBT cancer survivors and HCU barriers and psychosocial factors. Bivariate analyses were calculated to describe associations between HCU barriers, psychosocial factors, and sexual orientation and gender identity. To assess associations between HCU barriers and psychosocial factors, a Wald’s Chi-Square test and one-way ANOVA were conducted for each measure across LGBT groups. Alpha was set at 0.05.

### 3.6. Cluster Analysis

An agglomerative hierarchical cluster analysis was calculated with the 21 binary HCU barrier variables using Ward’s method with Euclidean distance [42]. The 21 HCU barrier variables used in the cluster analyses had binary responses (0 = No, 1 = Yes). Standardization was not applied to clustering because the variables were measured on the same scale and assumed to contribute equally to the distance metric. This approach is consistent with standard practice for clustering binary variables of equal weight and meaning [43]. The number of clusters was determined based on inspecting the dendrogram, pseudo-F statistic, and Cubic Clustering Criterion (CCC), consistent with established cluster analysis methods [42,44].

### 3.7. Cluster Psychosocial Characteristics

Psychosocial characteristics for each of the clusters and LGBT groups were described by calculating ANOVA with Tukey’s HSD post hoc tests to identify meaningful differences. Due to the privacy policies of the All of Us Research Program [45], results for groups with less than 20 observations per cell are not reported. All analyses were conducted using SAS 9.4 [46] within the All of Us Researcher Workbench [29].

### 3.8. Multinomial Logistic Regression

To examine demographic, socioeconomic, and psychosocial predictors of HCU barrier cluster membership, we estimated a multinomial logistic regression model using generalized logit specifications. Cluster membership, derived from prior cluster analysis of multidimensional HCU barriers, served as the nominal outcome variable with three mutually exclusive categories: low, moderate, and high HCU barriers. The low-barrier cluster was specified as the reference category for all comparisons.

The multinomial model simultaneously estimated the log odds of membership in the moderate- and high-barrier clusters relative to the low-barrier cluster. Analyses were conducted using PROC LOGISTIC in SAS with a generalized logit link function. Odds ratios (ORs) and 95% Wald confidence intervals (CIs) were computed for all predictors.

#### 3.8.1. Predictor Variables

Covariates were selected a priori based on theoretical relevance and prior literature on healthcare access and minority stress among sexual and gender minority cancer survivors. Predictors included age at analysis (continuous), age group at cancer diagnosis (0–17 years, 18–64 years, and ≥65 years), sex assigned at birth, sexual orientation, gender identity, race, income (continuous), everyday discrimination score (continuous), perceived stress score (continuous), social support score, and quality of life score (continuous). Categorical variables were parameterized using reference coding.

Sexual orientation was modeled with bisexual survivors specified as the reference group. This decision was theory- and data-driven. Results from the preceding cluster analysis demonstrated that bisexual cancer survivors were disproportionately represented in the high-barrier cluster and exhibited the highest levels of discrimination, perceived stress, and healthcare access challenges, as well as lower social support relative to other sexual orientation groups. Using bisexual survivors as the reference group therefore enabled direct estimation of relative advantage or disadvantage for lesbian and gay survivors compared with the subgroup experiencing the greatest cumulative HCU burden.

All reference categories were standardized to represent the most structurally disadvantaged groups based on theory, prior literature, and cluster membership frequencies by group. The sole exception was sex assigned at birth, for which the intersex category could not be used as a reference due to small sample size and resulting limitations in interpretability. Gender identity was modeled as not TGE vs. TGE (reference). Race was categorized as White, Black, and all other racial identities (reference). Educational attainment was included as a categorical variable with some college as the reference category.

#### 3.8.2. Model Estimation and Diagnostics

All models were estimated using maximum likelihood methods. Model convergence was assessed, and Wald confidence intervals were used to quantify uncertainty around effect estimates. Lack-of-fit diagnostics were requested to evaluate overall model adequacy. Predicted probabilities of cluster membership were generated at the individual level to support post-estimation interpretation and visualization of subgroup risk profiles. This analytic approach allowed for simultaneous evaluation of structural, socioeconomic, and psychosocial correlates of HCU barrier severity while preserving the nominal nature of the cluster outcome and enabling clear interpretation of subgroup differences across barrier levels.

## 4. Results

### 4.1. Sample Characteristics

Table 1 summarizes the demographic and health characteristics of LGBT cancer survivors across identity groups. TGE and bisexual survivors were notably younger at the time of analysis, with mean ages of 52.5 (SD = 17.3) and 55.7 (SD = 16.1) years, respectively, compared to gay (64.7, SD = 12.0) and lesbian (62.9, SD = 12.5) survivors. Across self-rated health measures, bisexual and TGE respondents reported lower perceived health and quality of life than their lesbian and gay peers. For example, TGE survivors had the lowest mean ratings for quality of life (1.81, SD = 1.08), physical health (1.70, SD = 1.15), and mental health (1.92, SD = 1.13), while gay and lesbian survivors reported higher values across these domains.

Sex assigned at birth varied substantially across sexual orientation and gender identity groups. Lesbian survivors were predominantly assigned female at birth (96.2%), whereas gay survivors were predominantly assigned male at birth (96.2%). In contrast, bisexual and TGE survivors demonstrated greater heterogeneity: 70.8% of bisexual survivors and 61.7% of TGE survivors were assigned female at birth, while 28.3% of bisexual and 34.9% of TGE survivors were assigned male at birth. Categories representing intersex or nonresponse were rare and suppressed due to small cell sizes.

Most respondents identified as White (82%), with racial minority representation highest among TGE individuals (15.8%). Education levels varied by group: 46.2% of lesbian and 37.1% of gay survivors had advanced degrees, compared to 34% of TGE and 32.3% of bisexual survivors. Bisexual and TGE individuals had the highest proportion of respondents with only some college education (28.9% and 29.7%, respectively). Cancer type distributions revealed striking differences. Skin (42.8%) and breast (12.5%) cancers were the most reported cancer types overall. Significant differences in cancer type emerged across subgroups, though low-frequency categories (e.g., lung, gastrointestinal, and sensory cancers) were not interpretable due to small cell sizes (*n* < 20). Skin cancer was most common among gay survivors (52.9%), followed by bisexuals (37.7%) and lesbians (27.5%). Breast cancer was most prevalent among lesbians (28.8%), with far lower prevalence among gay and TGE individuals. These subgroup differences highlight important variation in age, education, and cancer type distributions within LGBT cancer survivors, factors that may shape healthcare needs and utilization independent of sexual orientation or gender identity.

### 4.2. Healthcare Utilization Barriers and Psychosocial Characteristics

Table 2 presents descriptive comparisons of healthcare utilization barriers and cost-related behaviors across LGBT cancer survivors. Overall, bisexual and TGE respondents reported a higher prevalence of access barriers and unmet healthcare needs in the past 12 months compared with lesbian and gay respondents.

Delays in care related to transportation were more commonly reported by bisexual (15.2%) and TGE (21.4%) survivors than by lesbian (5.6%) and gay (8.0%) survivors. Similarly, inability to take time off work was more frequently reported among TGE (20.9%) and bisexual (11.6%) respondents relative to lesbian and gay respondents.

Cost-related barriers were prevalent across all groups but were notably more common among bisexual and TGE survivors. Nearly 30% of TGE survivors and 21.5% of bisexual survivors reported delaying care due to out-of-pocket costs, compared with approximately 13% among lesbian and gay survivors. Inability to afford prescription medications was also more frequently reported among TGE (29.4%) and bisexual (19.9%) respondents than among lesbian (8.7%) and gay (11.6%) respondents.

TGE survivors additionally reported higher levels of unmet need for mental health care (25.4%), dental care (30.4%), eyeglasses (31.3%), and specialty care (22.9%) compared with other groups. Cost-related medication behaviors followed a similar pattern, with TGE and bisexual respondents more frequently reporting delayed prescription fills, skipped doses, and requests for lower-cost medications.

Several items, including childcare-related barriers and caregiving responsibilities for another adult, were reported by relatively few respondents in certain groups and are therefore interpreted cautiously. Taken together, these descriptive patterns indicate a disproportionate burden of financial and structural healthcare barriers among bisexual and TGE cancer survivors.

Chi-square analyses revealed that bisexual and TGE survivors reported significantly higher rates of delayed care and cost-related barriers. Notable disparities included:

- Emergency care avoidance: TGE (11%) and bisexual (6.6%) vs. lesbian (2.7%);
- Prescription rationing behaviors: TGE individuals had higher rates of skipping doses, delaying fills, or asking for lower-cost options;
- Structural delays: TGE participants more frequently reported barriers like lack of childcare, transportation, and inability to afford out-of-pocket expenses.

### 4.3. Psychosocial Characteristics

As shown in Table 3, significant differences were observed across LGBT identity groups for composite measures of daily discrimination and perceived stress, with TGE and bisexual cancer survivors reporting higher scores than lesbian and gay survivors. On the discrimination composite, TGE survivors had the highest mean score (16.28, SD = 9.59), followed by bisexual survivors (11.61, SD = 8.98), while lesbian and gay survivors reported substantially lower values (7.46, SD = 6.44 and 7.82, SD = 6.93, respectively). Item-level responses reflect these patterns, with TGE respondents reporting the highest mean frequency for nearly all discrimination items, including being threatened or harassed (m = 1.31, SD = 1.31), being treated with less respect (m = 2.26, SD = 1.41), and being perceived as dishonest (m = 1.29, SD = 1.49). Bisexual respondents also reported elevated frequencies of mistreatment compared to lesbian and gay participants, while lesbians generally reported the lowest levels of daily discrimination experiences.

Composite perceived stress scores followed a similar pattern. TGE survivors reported the highest mean perceived stress (20.37, SD = 7.18), followed by bisexual survivors (18.58, SD = 8.29), whereas lesbian and gay survivors reported lower values (13.15, SD = 7.53 and 13.62, SD = 8.09, respectively). Item-level responses reflected these differences, with TGE survivors more frequently endorsing feelings of nervousness or stress (m = 2.55, SD = 1.14), lack of control over important things (m = 2.11, SD = 1.15), and inability to cope with demands (m = 2.01, SD = 1.07). Bisexual respondents showed similar elevated perceived stress profiles compared to lesbian and gay respondents.

Differences in composite social support scores across LGBT identity groups were statistically significant, but modest in magnitude. Lesbian survivors reported the highest overall social support (62.15, SD = 10.68), while bisexual (61.37, SD = 11.54), TGE (59.44, SD = 11.69), and gay (58.32, SD = 12.72) survivors reported slightly lower scores, with the overall range spanning approximately four points on the standardized scale. Item-level social support measures indicated that lesbian respondents more frequently reported access to tangible and emotional support, such as assistance if confined to bed, transportation to medical appointments, and help with daily chores. TGE respondents reported lower average scores across several support domains and higher reports of isolation-related items, including lack of companionship and feeling isolated. Bisexual respondents also reported higher feelings of isolation relative to lesbian respondents, though item-level differences across groups were small.

Overall, pronounced and consistent differences were observed across LGBT identity groups for daily discrimination and perceived stress, whereas variation in social support was comparatively limited. Item-level findings suggest differences in specific dimensions of perceived social support and social isolation, while composite social support scores indicate broadly similar levels of overall perceived support across groups.

**Figure 1:**
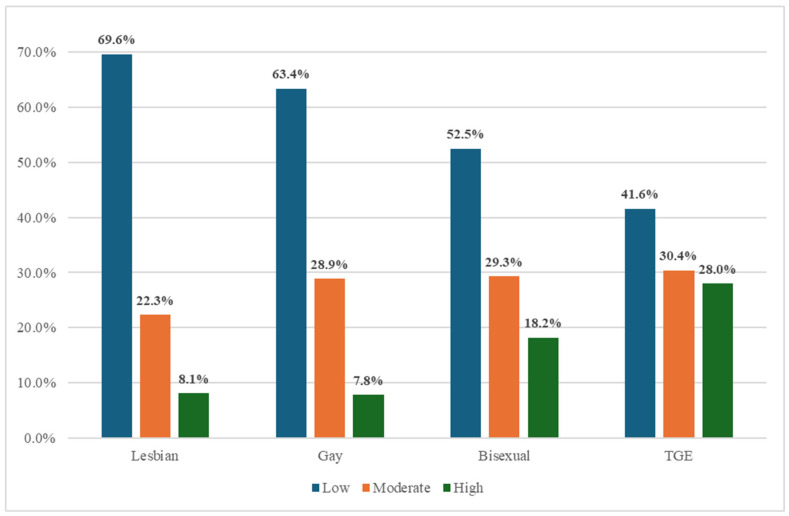
Percentage of LGBT Identity Across HCU Barrier Clusters.

Healthcare Utilization Barrier Clusters: A three-cluster solution emerged from the hierarchical cluster analysis, and Figure 1 illustrates the composition of cancer survivors in each cluster. Cluster 1 is characterized by cancer survivors who experience low healthcare utilization barriers. In cluster 1, the healthcare utilization means across (mostly <0.02) reflected minimal barriers in healthcare access or affordability. Most respondents (59.7%) across all LGBT groups fall in this cluster, including 69.6% of lesbians, 63.4% of gays, 52.5% of bisexuals, and 41.6% of TGE cancer survivors. Lesbian and gay participants were found to be the most likely to experience low barriers, while transgender and bisexual individuals are less likely to be included in the low-barrier group.

Cluster 2 is characterized by cancer survivors who experience moderate healthcare utilization barriers. In this cluster, the mean scores across healthcare access and affordability variables were moderate (generally ranging from 0.10 to 0.30), indicating a mid-level burden. The most notable barriers were struggling to afford eyeglasses (mean = 0.13), requesting lower-cost medications (mean = 0.22), and delaying care due to high deductibles (mean = 0.31). This group reflects a pattern of specific affordability-related challenges. The proportion of survivors in Cluster 2 was similar across LGBT subgroups: 22.3% of lesbians, 28.0% of gay individuals, 29.3% of bisexuals, and 30.4% of transgender individuals fell into this moderate-barrier group. Although all groups are represented, bisexual and transgender cancer survivors appear slightly more likely to face moderate barriers.

Cluster 3 is characterized by cancer survivors who experience high healthcare utilization barriers. The mean scores across most variables were substantially elevated, indicating widespread and severe difficulties accessing care. For example, the mean for being unable to afford prescriptions was 0.77, for dental care was 0.79, for delaying prescription fills was 0.72, and for delaying care due to out-of-pocket costs was 0.70. Many other indicators in this cluster had means exceeding 0.60, underscoring the intensity of financial and access-related hardship. This high-barrier group includes a disproportionate number of bisexual (18.2%) and transgender (28.0%) cancer survivors, with notably lower representation from lesbian (8.1%) and gay (7.8%) individuals. These findings highlight a concentration of high healthcare barriers among bisexual and transgender survivors, pointing to pronounced disparities that warrant focused policy and clinical attention.

### 4.4. Psychosocial Characteristics and Health Utilization Barrier Clusters

As summarized in Table 4, psychosocial characteristics varied significantly across the three HCU barrier clusters. The average daily discrimination scores were highest in High Barriers Cluster (m = 16.20, SD = 10.57) and lowest in the Low Barriers cluster (m = 7.5, SD = 7.29). Perceived stress scores followed a similar pattern, increasing across clusters. Participants in the high barriers cluster also reported the highest average perceived stress (m = 22.23, SD = 7.45), while the low barriers cluster reported the lowest (m = 13.4, SD = 7.85) average perceived stress.

Conversely, differences in social support scores across HCU clusters were modest. While mean social support was slightly higher in the low-barrier cluster (m = 60.33, SD = 11.72) and lower in the high-barrier cluster (m = 58.63, SD = 13.11), these differences were small in magnitude and did not significantly differentiate cluster membership (F = 2.67). This pattern suggests that, unlike discrimination and perceived stress, overall perceived social support did not vary meaningfully across levels of HCU burden in this sample.

### 4.5. Multinomial Logistic Regression Predicting Healthcare Utilization Barrier Clusters

Table 5 presents results from the multinomial logistic regression examining demographic, socioeconomic, and psychosocial correlates of membership in the moderate- and high–HCU barrier clusters relative to the low-barrier reference group. Several factors were independently associated with elevated odds of high-barrier cluster membership, while others showed limited or no association after multivariable adjustment.

Sexual orientation remained a consistent and independent predictor of HCU burden. Compared with bisexual survivors, lesbian survivors had significantly lower odds of moderate-barrier membership (AOR = 0.55, 95% CI: 0.40–0.75) and high-barrier membership (AOR = 0.58, 95% CI: 0.36–0.94). No statistically significant differences were observed for gay survivors relative to bisexual survivors. Estimates for individuals identifying as straight or selecting another non-listed sexual orientation were imprecise, with wide confidence intervals reflecting small cell sizes, and should be interpreted cautiously. These findings reinforce descriptive patterns indicating that bisexual survivors are disproportionately represented among those experiencing greater structural and cost-related barriers to care.

Sex assigned at birth was independently associated with HCU barrier membership. Compared with survivors assigned female at birth, those assigned male at birth had significantly lower odds of membership in both the moderate- (AOR = 0.65, 95% CI: 0.46–0.93) and high-barrier clusters (AOR = 0.53, 95% CI: 0.30–0.93). Estimates for individuals classified as intersex, none of these, or nonresponse were imprecise and not statistically significant due to small cell sizes.

Income demonstrated a strong and specific association with high-barrier status. Higher income was associated with substantially lower odds of membership in the high-barrier cluster (AOR = 0.82, 95% CI: 0.75–0.90), reflecting a pronounced socioeconomic gradient in severe HCU burden. In contrast, income was not significantly associated with membership in the moderate-barrier cluster, suggesting that financial resources most clearly differentiate survivors facing the most intensive access challenges rather than intermediate levels of burden.

Psychosocial stressors emerged as some of the most robust predictors of cluster membership. Each one-unit increase in everyday discrimination score was associated with higher odds of belonging to both the moderate-barrier (AOR = 1.03, 95% CI: 1.01–1.04) and high-barrier clusters (AOR = 1.04, 95% CI: 1.02–1.06). Perceived stress showed a graded association with HCU burden and was significantly associated with high-barrier membership (AOR = 1.06, 95% CI: 1.03–1.09), while its association with moderate-barrier membership did not reach statistical significance. In contrast, social support did not independently predict cluster membership. After adjustment for discrimination, stress, income, and other covariates, social support coefficients were near null for both moderate- (AOR = 1.00, 95% CI: 0.99–1.01) and high-barrier clusters (AOR = 0.99, 95% CI: 0.98–1.01). This suggests that while social support varies descriptively across identity groups and clusters, it does not independently offset the effects of structural and psychosocial stressors driving HCU burden.

Gender identity was not significantly associated with HCU cluster membership. Compared with TGE survivors, non-TGE survivors had lower point estimates for both moderate- (AOR = 0.68) and high-barrier cluster membership (AOR = 0.70), though confidence intervals were wide and included the null. Age at analysis was inversely associated with high-barrier membership (AOR = 0.98, 95% CI: 0.96–0.99), indicating lower odds of severe HCU burden among older survivors. Age group at diagnosis was not consistently associated with cluster membership after adjustment, although survivors diagnosed at age 65 years or older had lower odds of membership in the high-barrier cluster compared with those diagnosed before age 18 (AOR = 0.24, 95% CI: 0.08–0.77). Neither race nor education exhibited statistically significant associations with HCU cluster membership.

Overall, the regression results identify a concentrated pattern of vulnerability characterized by bisexual identity, female sex assigned at birth, lower income, and elevated discrimination and stress. These factors remained strong predictors of HCU burden after multivariable adjustment, underscoring a healthcare access landscape shaped less by interpersonal resources and more by cumulative structural and psychosocial disadvantage among LGBT cancer survivors.

## 5. Discussion

This study offers new insights into the healthcare utilization (HCU) barriers and psychosocial determinants among LGBT cancer survivors using national data from the All of Us Research Program. To our knowledge, this is the first study to apply cluster analysis to identify survivor typologies based on multidimensional HCU burden. Findings revealed substantial heterogeneity in survivorship experiences across sexual orientations and gender identity, underscoring the disproportionate vulnerability of bisexual and TGE survivors. Interpretation of these findings should emphasize structural and social context rather than identity-based causation. The observed differences may reflect differential burden within existing cancer survivorship care systems, rather than evidence that LGBTQ+ identity itself causes healthcare utilization barriers. Many of the barriers observed are likely manifestations of broader systemic challenges in survivorship care, including affordability, access, and administrative complexity. Accordingly, this analysis focuses on how shared structural barriers are differentially experienced across LGBT subgroups, rather than attributing disparities exclusively to sexual orientation or gender identity. These structural dynamics also shape who is represented in large national research cohorts.

The AoU research program is committed to enrolling racially and ethnically diverse participants [47]. Yet, this may be challenged due to historic [48] and persisting [49] unethical treatment of racial/ethnic minoritized groups in medical settings. Unethical and harmful treatment in medical settings may contribute to lower rates of participation in health surveillance and research activities among diverse racial and ethnic groups.

Our results confirm and extend prior work documenting elevated healthcare utilization barriers among sexual and gender minorities [7,8]. Bisexual and TGE survivors consistently reported greater affordability-related and structural barriers, particularly around prescription costs, transportation, and workplace flexibility, than their lesbian and gay counterparts. These patterns reflect broader systemic inequities, aligning with minority stress theory, which posits that cumulative exposure to stigma and discrimination exacerbates stress and constrains healthcare engagement among sexual and gender minority populations [18].

Differences in cancer type distribution across LGBT subgroups likely reflect socially patterned risk exposures and sex assigned at birth-related biology rather than identity-specific susceptibility. The higher prevalence of skin cancer among gay survivors is consistent with prior studies documenting elevated indoor tanning and ultraviolet exposure among sexual minority men, behaviors shaped by sociocultural norms around appearance and body image and known to increase melanoma and keratinocyte cancer risk [50,51,52]. In contrast, variation in breast cancer prevalence largely tracks sex assigned at birth and cumulative hormone exposure. Evidence indicates that breast cancer risk is highest among individuals assigned female at birth, while transgender women receiving feminizing hormone therapy have intermediate risk relative to cisgender men and women [53,54]. Additionally, sexual minority women and TGE individuals experience differences in reproductive risk factors and barriers to routine cancer screening, which may further influence observed prevalence [55].

Observed differences in healthcare utilization barriers across LGBT subgroups may reflect variation in age distributions, racial composition, and gender identity; however, this analysis did not apply formal intersectional methods capable of fully capturing overlapping identities. In multinomial models adjusting for age, race, income, education, and marital status, sexual orientation remained a consistent independent predictor of HCU burden, with bisexual cancer survivors showing disproportionately higher odds of membership in both moderate- and high-barrier clusters. Income emerged as a strong protective factor against high-barrier membership, highlighting a pronounced socioeconomic gradient in severe healthcare access challenges.

The associations observed for income and education in the adjusted models warrant clarification. Income showed a strong association with healthcare utilization barrier severity, with higher income associated with lower odds of membership in the high-barrier cluster. In contrast, relative to individuals with some college education or more, those with a high school education or less showed directionally lower odds of high-barrier cluster membership, while individuals with college or advanced degrees showed directionally higher odds; however, all education estimates were imprecise, and confidence intervals crossed the null. These patterns suggest that educational attainment in this context may capture differences in healthcare system engagement, expectations, or exposure to administrative complexity rather than functioning as a marker of material resources.

Gender identity showed elevated but non-significant associations for transgender and gender-expansive survivors, likely reflecting limited precision due to smaller subgroup sizes. Race, education, and marital status did not demonstrate consistent independent associations after adjustment, despite descriptive differences across subgroups. Taken together, these findings indicate that sexual orientation, income, and cumulative psychosocial stressors were the most consistent predictors of healthcare utilization barriers, outweighing demographic composition alone in shaping survivorship access patterns among LGBT cancer survivors.

The three-cluster typology (low, moderate, and high HCU barrier groups) highlights distinct survivor profiles. Lesbian and gay participants predominated in the low-barrier cluster, whereas bisexual and TGE survivors were concentrated in the high-barrier group, characterized by extensive cost-related and logistical obstacles. This gradient of disadvantage parallels observed disparities in perceived discrimination, stress, and social support [15,56], emphasizing how psychosocial adversity and structural exclusion operate synergistically to restrict survivorship care engagement.

Consistent with previous studies linking discrimination and chronic stress to adverse health outcomes [19,57], psychosocial indicators in this sample appeared to have followed a dose–response pattern, with higher discrimination and perceived stress corresponding to greater HCU barriers. In contrast, variation in overall perceived social support across HCU clusters was limited. Although bisexual and TGE survivors reported higher endorsement of isolation-related items and lower availability of certain forms of tangible support, composite social support scores differed modestly across groups and were not independently associated with cluster membership in adjusted analyses. These findings suggest that social support may function as a relatively stable background resource that is insufficient to offset the effects of sustained structural and psychosocial strain, rather than as a primary driver of HCU barriers. While social support was descriptively associated with lower HCU burden, it did not attenuate the strong associations observed for discrimination and perceived stress. This pattern is consistent with prior psychometric work demonstrating that multidimensional functional support measures capture availability rather than adequacy or contextual fit of support, which could limit their sensitivity to structural barriers in survivorship care [38,58].

Future research should prioritize longitudinal and mixed-methods designs to clarify the temporal ordering between psychosocial stressors and healthcare utilization barriers among LGBT cancer survivors. Linking repeated measures of discrimination, stress, and social support with clinical, pharmacy, and insurance data would allow investigators to distinguish whether psychosocial adversity drives disengagement from care or whether persistent structural barriers erode support networks and exacerbate stress over time. Such approaches are critical for identifying intervention points that effectively disrupt these reinforcing cycles.

### 5.1. Clinical and Policy Implications

These findings carry several actionable implications. Clinically, integrating systematic screening for affordability and structural barriers, perceived discrimination, and psychosocial stressors into survivorship care—whether delivered through formal survivorship care plans, survivorship navigation programs, or routine follow-up workflows—may help identify at-risk patients earlier in their care trajectory. The high concentration of bisexual and TGE survivors in the high-barrier cluster underscores the need for tailored interventions addressing cost, transportation, and workplace-related constraints, alongside affirming provider training that targets implicit bias and misgendering.

LGBTQ+ cancer survivors are a diverse and growing population; in 2024 the American Cancer Society [3] estimated that 160,000 LGBTQ + people were diagnosed with cancer and nearly 50,000 would die from cancer. The National Cancer Institute [59], American Society of Clinical Oncology (ASCO) [60], The American Cancer Society [3], the National Comprehensive Network [61] and others [60,62] all recommend regular and systematic SOGI data collection in cancer care. In their 2022, report the National Academies demonstrated the feasibility and acceptability of SOGI data collection by patients [63]. SOGI data collection is a foundational requirement for monitoring LGBTQ+ cancer survivors’ unique needs and experiences in oncology practice and research [59,60]. SOGI data collection is also vital to building trust and rapport between patients and providers [59] and for ensuring every patient receives the most appropriate care and treatment.

When implemented transparently and with appropriate safeguards, SOGI data collection has been shown to support patient-provider communication, strengthen rapport, and enhance trust by signaling recognition, inclusion, and respect for patients’ identities [59,64]. However, to avoid the risk of exacerbating mistrust or discrimination, SOGI data collection must occur in concert with robust governance systems, including explicit anti-discrimination policies and enforcement mechanisms, use of validated and best-practice SOGI measures, health system safeguards, mandatory and ongoing cultural humility training for providers and staff, and clear patient communication regarding the purpose of SOGI data collection and the associated privacy and confidentiality protections [59,65]. Without these conditions, SOGI data collection may undermine patient willingness to disclose sensitive information and care engagement. Due to the data available in the AoU research program, these data were limited to LGBT identifying cancer survivors. However, efforts to address inclusion and equity in cancer care must be inclusive of the many diverse identities under the LGBTQ+ umbrella. For example, it is critical that cultural humility education, sexual orientation and gender identity (SOGI) data collection in the health record, nondiscrimination policies visible to providers, staff, and patients, and survivor navigation programs be inclusive of all LGBTQ+ identities, not only LGBT identities.

At the systems level, health policies should expand financial protection mechanisms to mitigate cost-related nonadherence and promote equitable insurance coverage. Efforts to embed LGBTQ+ cultural competence within oncology care, through inclusive electronic health record documentation, visible nondiscrimination policies, and survivor navigation programs, are critical. Future survivorship frameworks must explicitly account for the heterogeneity within the LGBT umbrella to ensure bisexual and TGE survivors receive affirming, continuous, and affordable care.

### 5.2. Limitations

Several limitations should be considered. The cross-sectional design limits causal inference regarding relationships between psychosocial factors and healthcare utilization. Next, all measures—including cancer history—were self-reported, introducing potential recall or social desirability bias. However, prior validation studies in large U.S. cohort studies demonstrate moderate to high concordance between self-reported cancer diagnoses and cancer registry data, particularly for common cancers such as breast, prostate, melanoma, and colorectal cancer, suggesting that self-reported cancer history is generally reliable in population-based research [66,67,68]. When misclassification occurs, evidence indicates it is more likely due to underreporting rather than false-positive reporting of cancer diagnoses [66,67,69,70]. Additionally, survivorship timing could not be modeled directly because age at diagnosis was not available as a continuous variable in the survey data. The only diagnosis-related timing measure available was age group at diagnosis, reported in broad categorical bands (e.g., 0–11, 12–17, 18–64, ≥65 years). As a result, time since diagnosis could not be calculated reliably by subtracting age group from continuous age at the time of survey, as doing so would yield imprecise and potentially misleading estimates. To partially account for survivorship timing, age group at diagnosis was included as a covariate in descriptive analyses and multivariable models.

Small subgroup sample sizes due to All of Us data suppression rules constrained the examination of intersectional subpopulations (e.g., racial/ethnic minorities within TGE survivors). While the All of Us cohort is demographically diverse, it is not nationally representative, which may limit generalizability. Additionally, medication-related healthcare utilization barriers were assessed using binary, self-reported items, which do not capture the underlying reasons for nonadherence. As a result, we were unable to distinguish whether reported behaviors reflected financial barriers, experiences of discrimination, concerns about medication interactions (including with gender-affirming therapies), or other clinical or contextual factors, particularly among TGE survivors.

Cluster analytic techniques are also exploratory and sensitive to methodological decisions, including distance metrics and the number of clusters retained. Finally, we did not adjust for cancer-specific variables (e.g., stage, treatment type, comorbidities), which may independently influence healthcare access and psychosocial outcomes.

## 6. Conclusions

This analysis advances understanding of disparities in cancer survivorship by elucidating distinct HCU barrier typologies among LGBT survivors. Bisexual and TGE individuals face disproportionate structural and psychosocial burdens, including cost-related care delays, discrimination, and isolation-related support gaps. These findings underscore the necessity of developing survivor-centered care models that integrate screening for discrimination, chronic stress, and structural barriers, alongside provider education, and structural reforms to reduce financial toxicity and access inequities. Future research should evaluate longitudinal outcomes and intervention effectiveness within these high-risk subgroups to promote equitable, affirming survivorship care across the cancer continuum.

## Figures and Tables

**Table 1:** Sample Demographic and Healthcare Characteristics.

		Lesbian (*n* = 730)	Gay (*n* = 1285)	Bisexual (*n* = 1296)	TGE (*n* = 209)	*p*-Values
Characteristic		*N* (%) or Mean (SD)				
**AGE**	Age at time of analysis	62.9 (12.5)	64.7 (12.0)	55.7 (16.1)	52.5 (17.3)	*p* < 0.0001
**SELF-RATED HEALTH**	In general, would you say your health is:	2.22 (1.00)	2.25 (0.97)	1.96 (1.03)	2.09 (1.06)	*p* < 0.0001
	In general, would you say your quality of life is:	2.75 (0.97)	2.66 (1.02)	2.33 (1.06)	1.81 (1.08)	*p* < 0.0001
	In general, how would you rate your physical health?	2.15 (1.00)	2.18 (0.97)	1.86 (1.04)	1.70 (1.15)	*p* < 0.0001
	In general, how would you rate your mental health, including your mood and your ability to think?	2.62 (0.98)	2.60 (1.06)	2.17 (1.12)	1.92 (1.13)	*p* < 0.0001
**RACE**	White	666 (91.2%)	1117 (86.9%)	1118 (86.3%)	176 (84.2%)	*p* = 0.004
	Non-White or skip	64 (8.8%)	158 (13.1%)	178 (13.7%)	33 (15.8%)	*p* = 0.16
**EDUCATION**	High School or Lower or Skip	42 (5.8%)	94 (7.3%)	158 (12.2%)	20 (9.6%)	*p* < 0.0001
	Some College (1–3)	149 (20.4%)	296 (23.0%)	374 (28.9%)	62 (29.7%)	*p* < 0.0001
	College Grad	195 (26.7%)	408 (31.8%)	331 (25.5%)	53 (25.4%)	*p* < 0.0001
	Advanced Degree	337 (46.2%)	477 (37.1%)	419 (32.3%)	71 (34.0%)	*p* < 0.0001
**ETHNICITY**	Latino/Hispanic	23 (3.2%)	59 (4.7%)	47 (3.8%)	**	*p* = 0.16
**SEX AT BIRTH**	Female	702 (96.2%)	43 (3.4%)	918 (70.8%)	129 (61.7%)	*p* < 0.0001
	Male	24 (3.3%)	1236 (96.2%)	367 (28.3%)	73 (34.9%)	*p* < 0.0001
	Intersex/None of These/Prefer Not/Skip	**	**	**	**	**
**CANCER TYPE**	Skin Cancer	275 (27.5%)	680 (52.9%)	488 (37.7%)	67 (32.1%)	*p* < 0.0001
	Sex-Specific Cancers (Prostate, Cervical, Ovarian, or Endometrial) *	97 (2.8%)	257 (20%)	139 (10.7%)	31 (14.8%)	*p* < 0.0001
	Breast Cancer	210 (28.8%)	**	187 (14.4%)	29 (13.9%)	*p* < 0.0001
	Blood, Bone, Endocrine, and Soft Tissue Cancers *	55 (7.5%)	130 (10.1%)	115 (8.9%)	29 (13.9%)	*p* = 0.028
	Lung Cancer	**	22 (1.7%)	**	**	*p* = 0.19
	Gastrointestinal Cancers	24 (3.3%)	111 (8.6%)	68 (5.3%)	**	*p* < 0.0001
	Head, Neck, and Sensory Cancers	**	47 (1.3%)	37 (3.9%)	**	*p* = 0.27
	Other (specified) Cancers *	55 (7.5%)	157 (12.2%)	105 (8.1%)	22 (10.5%)	*p* < 0.0001
**AGE AT CANCER DIAGNOSIS**	Child (0 to 11 years old)	**	**	22 (1.7%)	**	*p* < 0.0001
	Adolescent (12 to 17 years old)	**	23 (1.8%)	64 (4.9%)	**	*p* < 0.0001
	Adult (18 to 64 years old)	610 (83.6%)	1011 (78.7%)	1051 (81.1%)	158 (75.6%)	*p* = 0.015
	Older Adult (65 to 75 years old)	94 (12.9%)	218 (17.0%)	134 (10.3%)	24 (11.5%)	*p* < 0.0001
	Elderly (75+ years old)	**	23 (1.8%)	25 (1.9%)	**	*p* = 0.43

* = combined due to cells ≤ 20; ** = data redacted due to cell size ≤ 20.

**Table 2:** LGBT Cancer Survivors’ Reasons for Healthcare Delays.

		Lesbian	Gay	Bisexual	TGE	*p*-Value
Social Determinants of Health		*N* (% Yes)				
There are many reasons people delay getting medical care. Have you delayed getting care for any of the following reasons in the PAST 12 MONTHS? (*n*/% yes)	Didn’t have transportation.	40 (5.6%)	100 (8.0%)	191 (15.2%)	43 (21.4%)	<0.0001
	Couldn’t get time off work.	44 (6.2%)	56 (4.5%)	145 (11.6%)	42 (20.9%)	<0.0001
	Couldn’t get childcare.	*	*	59 (4.7%)	*	<0.0001
	You provide care to an adult and cannot leave him/her.	*	*	51 (4.1%)	*	<0.0001
	Couldn’t afford the copay.	45 (6.4%)	71 (5.6%)	151 (12.0%)	40 (19.9%)	<0.0001
	Your deductible was too high or could not afford the deductible.	45 (6.4%)	80 (6.4%)	160 (12.8%)	36 (17.9%)	<0.0001
	You had to pay out of pocket for some or all of the procedure.	96 (13.5%)	161 (12.8%)	270 (21.5%)	60 (29.9%)	<0.0001
	Prescription medicines	62 (8.7%)	146 (11.6%)	249 (19.9%)	59 (29.4%)	<0.0001
	Mental health care or counseling	51 (7.2%)	90 (7.2%)	210 (16.8%)	51 (25.4%)	<0.0001
DURING THE PAST 12 MONTHS, was there any time when you needed any of the following, but didn’t get it because you couldn’t afford it?	Emergency care	*	37 (2.9%)	82 (6.5%)	22 (11%)	<0.0001
	Dental care (including checkups)	114 (16.1%)	170 (13.5%)	333 (26.6%)	61 (30.4%)	<0.0001
	Eyeglasses	72 (10.1%)	125 (9.9%)	268 (21.4%)	63 (31.3%)	<0.0001
	To see a regular doctor or general health provider (in primary care, general practice, internal medicine, family medicine)	21 (3.0%)	45 (3.6%)	115 (9.2%)	25 (12.4%)	<0.0001
	To see a specialist	53 (7.5%)	83 (6.6%)	185 (14.8%)	46 (22.9%)	<0.0001
	Follow-up care	29 (4.1%)	66 (5.3%)	155 (12.4%)	33 (16.4%)	<0.0001
	You skipped medication doses to save money	43 (6.1%)	91 (7.2%)	170 (13.6%)	44 (21.9%)	<0.0001
	You took less medicine to save money	42 (5.9%)	86 (6.8%)	196 (15.6%)	47 (23.4%)	<0.0001
	You delayed filling a prescription to save money	86 (12.1%)	124 (9.9%)	253 (20.2%)	60 (29.9%)	<0.0001
DURING THE PAST 12 MONTHS, were any of the following true for you?	You asked your doctor for a lower cost medication to save money	117 (16.5%)	259 (20.6%)	343 (27.4%)	71 (35.3%)	<0.0001
	You bought prescription drugs from another country to save money	*	69 (5.5%)	59 (4.7%)	*	0.0151
	You used alternative therapies to save money	47 (6.6%)	58 (4.6%)	186 (14.8%)	38 (18.9%)	<0.0001

* = data redacted due to cell ≤ 20.

**Table 3:** LGBT Cancer Survivors’ Psychosocial Factor Scores.

		Lesbian	Gay	Bisexual	TGE	*p*-Value
Psychosocial Factors		*N* (% Yes)				
In your day-to-day life… (0 = Never to 5 = Almost Everyday)	how often are you threatened or harassed?	0.57 (0.87)	0.65 (0.98)	0.87 (1.11)	1.31 (1.31)	<0.001
	how often are you called names or insulted?	0.66 (0.91)	0.78 (1.04)	0.98 (1.26)	1.38 (1.44)	<0.001
	how often do people act as if they are afraid of you?	0.50 (0.94)	0.65 (1.10)	0.73 (1.14)	1.37 (1.63)	<0.001
	how often do people act as if they’re better than you are?	1.29 (1.16)	1.29 (1.32)	1.73 (1.45)	2.38 (1.50)	<0.001
	how often do people act as if they think you are dishonest?	0.33 (0.74)	0.49 (0.92)	0.76 (1.14)	1.29 (1.49)	<0.001
	how often are you treated with less respect than other people?	1.32 (1.16)	1.21 (1.23)	1.54 (1.32)	2.26 (1.41)	<0.001
	how often do you receive poorer service than other people at restaurants or stores?	0.89 (0.97)	0.78 (1.05)	0.98 (1.13)	1.48 (1.33)	<0.001
	how often do people act as if they think you are not smart?	0.89 (1.11)	0.80 (1.19)	1.45 (1.47)	1.97 (1.60)	<0.001
	how often are you treated with less courtesy than other people?	1.31 (1.16)	1.22 (1.24)	1.50 (1.31)	2.26 (1.42)	<0.001
Perceived Stress (0 = Never to 5 = Very Often)	In the last month, how often have you been upset because of something that happened unexpectedly?	1.56 (0.92)	1.53 (1.01)	1.88 (1.02)	2.06 (1.06)	<0.001
	In the last month, how often have you felt that you were unable to control the important things in your life?	1.45 (1.10)	1.37 (1.10)	1.89 (1.18)	2.11 (1.15)	<0.001
	In the last month, how often have you felt nervous and “stressed”?	1.95 (1.04)	1.89 (1.14)	2.40 (1.16)	2.55 (1.14)	<0.001
	In the last month, how often have you felt confident about your ability to handle your personal problems? *	0.92 (0.98)	1.01 (1.10)	1.30 (1.06)	1.50 (1.09)	<0.001
	In the last month, how often have you felt that things were going your way? *	1.31 (1.01)	1.36 (1.02)	1.67 (1.05)	1.90 (0.99)	<0.001
	In the last month, how often have you found that you could not cope with all the things that you had to do?	1.32 (1.06)	1.25 (1.07)	1.72 (1.13)	2.01 (1.07)	<0.001
	In the last month, how often have you been able to control irritations in your life? *	1.16 (1.02)	1.16 (1.05)	1.45 (1.04)	1.56 (0.96)	<0.001
	In the last month, how often have you felt that you were on top of things? *	1.26 (1.04)	1.20 (0.98)	1.64 (1.10)	1.93 (1.03)	<0.001
	In the last month, how often have you been angered because of things that were outside of your control?	1.54 (0.99)	1.58 (1.02)	1.87 (1.09)	1.96 (1.05)	<0.001
Social Support (0 = None of the time, 4 = All of the Time; 0 = Never, 3 = Often)	Someone to help you if you were confined to bed	3.05 (1.14)	2.64 (1.36)	2.71 (1.25)	2.42 (1.36)	<0.001
	Someone to take you to the doctor if you need it	3.23 (1.03)	2.86 (1.26)	2.87 (1.22)	2.63 (1.33)	<0.001
	Someone to prepare your meals if you were unable to do it yourself	2.97 (1.22)	2.52 (1.43)	2.62 (1.33)	2.42 (1.39)	<0.001
	Someone to help with daily chores if you were sick	2.93 (1.27)	2.47 (1.44)	2.52 (1.36)	2.31 (1.43)	<0.001
	Someone to have a good time with	3.12 (1.06)	2.75 (1.18)	2.76 (1.18)	2.63 (1.24)	<0.001
	Someone to turn to for suggestions about how to deal with a personal problem	3.14 (1.09)	2.75 (1.23)	2.78 (1.20)	2.56 (1.26)	<0.001
	Someone who understands your problems	3.01 (1.06)	2.67 (1.21)	2.59 (1.21)	2.46 (1.17)	<0.001
	Someone to love and make you feel wanted	3.21 (1.15)	2.68 (1.39)	2.80 (1.29)	2.58 (1.38)	<0.001
	I lack companionship	1.01 (1.01)	1.40 (1.11)	1.40 (1.05)	1.64 (1.11)	<0.001
	There is no one I can turn to	0.63 (0.86)	0.90 (0.98)	0.99 (0.97)	1.20 (1.07)	<0.001
	I am an outgoing person	2.21 (0.73)	2.17 (0.78)	2.05 (0.84)	1.85 (0.87)	<0.001
	I feel left out	1.15 (0.88)	1.24 (0.93)	1.48 (0.91)	1.69 (1.00)	<0.001
	I feel isolated from others	1.03 (0.95)	1.27 (1.00)	1.47 (1.00)	1.72 (1.05)	<0.001
	I can find companionship when I want it	2.55 (0.76)	2.31 (0.83)	2.33 (0.81)	2.09 (0.90)	<0.001
	I am unhappy being so withdrawn	0.80 (0.89)	1.00 (0.98)	1.20 (1.01)	1.44 (1.07)	<0.001

* indicates value has been reverse coded (i.e., higher numbers indicate more stress).

**Table 4:** Average Psychosocial Scores Across LGBT Identities and HCU Clusters.

	Lesbian	Gay	Bisexual	TGE		Low	Moderate	High	
Psychosocial Measure	Mean (SD)				F-Value	Mean (SD)			F-Value
Daily Discrimination	7.46 (6.44)	7.82 (6.93)	11.61 (8.98)	16.28 (9.59)	62.16 *	7.46 (7.29)	9.95 (7.83)	16.20 (10.57)	142.42 *
Perceived Stress	13.15 (7.53)	13.62 (8.09)	18.58 (8.29)	20.37 (7.18)	63.22 *	13.24 (7.85)	15.77 (7.91)	22.23 (7.45)	159.07 *
Social Support	62.15 (10.68)	58.32 (12.72)	61.37 (11.54)	59.44 (11.69)	11.65 *	60.33 (11.72)	60.02 (12.02)	58.63 (13.11)	2.67

* *p*-value < 0.0001.

**Table 5:** Multinomial Logistic Regression Predicting Moderate and High HCU Barrier Cluster Membership (Ref = Low Barriers).

		Adjusted Odds Ratios (AORs) and 95% Confidence Intervals (95% CIs)	
Predictor	Comparison Group	Moderate vs. Low Barriers	High vs. Low Barriers
Age at analysis	Per 1-year increase	1.00 (0.99–1.02)	0.98 (0.96–0.99)
Age at diagnosis (ref. 0–17)	18–64	0.83 (0.46–1.48)	0.61 (0.33–1.14)
	65+	0.72 (0.36–1.45)	0.24 (0.08–0.77)
Sexual orientation (ref. Bisexual)	Gay	1.06 (0.75–1.50)	1.04 (0.57–1.89)
	Lesbian	0.55 (0.40–0.75)	0.58 (0.36–0.94)
	Straight	1.16 (0.64–2.12)	1.23 (0.56–2.72)
	Other/Not listed	1.03 (0.46–2.31)	1.37 (0.55–3.42)
Gender identity (ref. TGE)	Not TGE	0.68 (0.38–1.20)	0.70 (0.35–1.41)
Race (ref. All other races)	White	2.03 (0.98–4.20)	1.34 (0.50–3.58)
	Black	1.19 (0.51–2.82)	1.28 (0.43–3.86)
Education (ref. Some college)	Advanced degree	1.23 (0.90–1.68)	1.19 (0.76–1.89)
	High school or less	0.65 (0.39–1.06)	0.63 (0.36–1.10)
	College graduate	1.34 (0.98–1.82)	1.28 (0.84–1.96)
Sex assigned at birth (ref. Female)	Male	0.65 (0.46–0.93)	0.53 (0.30–0.93)
	Intersex/none/missing	0.66 (0.12–3.58)	3.20 (0.69–14.96)
Income	Per unit increase	0.99 (0.93–1.05)	0.82 (0.75–0.90)
Marital status (ref. Not married)	Living with partner	1.07 (0.76–1.50)	0.78 (0.48–1.27)
	Married	1.06 (0.81–1.38)	1.22 (0.82–1.81)
Discrimination score	Per unit increase	1.03 (1.01–1.04)	1.04 (1.02–1.06)
Perceived stress	Per unit increase	1.02 (1.00–1.03)	1.06 (1.03–1.09)
Social support	Per unit increase	1.00 (0.99–1.01)	0.99 (0.98–1.01)
Quality of life	Per unit increase	0.86 (0.76–0.99)	0.70 (0.58–0.84)

## Data Availability

Restrictions apply to the availability of the data. Data were obtained from the All of Us Research Program, Controlled Tier V8. The data are available to qualified researchers through the All of Us Workbench following approval of a data use agreement. Due to privacy protections and disclosure risk, particularly for small cell sizes, the data cannot be made publicly available.

## Correction Statement

Following the removal of this article from the Special Issue “Cancer Disparities and Health Equity: Challenges, Insights, and Transformative Strategies”, an Editorial Board member reviewed the entire editorial process and confirmed all elements had been carried out as per MDPI Editorial Process policy (https://www.mdpi.com/editorial_process). As a result, the original Academic Editor listed on this publication has been replaced with that of the Editorial Board member who conducted this review. The original publication has now been updated accordingly. This information has also been transmitted to relevant indexing organizations.
